# One-step generation of a targeted knock-in calf using the CRISPR-Cas9 system in bovine zygotes

**DOI:** 10.1186/s12864-021-07418-3

**Published:** 2021-02-12

**Authors:** Joseph R. Owen, Sadie L. Hennig, Bret R. McNabb, Tamer A. Mansour, Justin M. Smith, Jason C. Lin, Amy E. Young, Josephine F. Trott, James D. Murray, Mary E. Delany, Pablo J. Ross, Alison L. Van Eenennaam

**Affiliations:** 1 Department of Animal Science, University of California – Davis, Davis, CA USA; 2 Department of Population Health and Reproduction, School of Veterinary Medicine, University of California – Davis, Davis, CA USA; 3 Department of Clinical Pathology, School of Medicine, University of Mansoura, Mansoura, Egypt

**Keywords:** CRISPR, Knock-in, Gene editing, Bovine, Embryos, *Bos taurus*

## Abstract

**Background:**

The homologous recombination (HR) pathway is largely inactive in early embryos prior to the first cell division, making it difficult to achieve targeted gene knock-ins. The homology-mediated end joining (HMEJ)-based strategy has been shown to increase knock-in efficiency relative to HR, non-homologous end joining (NHEJ), and microhomology-mediated end joining (MMEJ) strategies in non-dividing cells.

**Results:**

By introducing gRNA/Cas9 ribonucleoprotein complex and a HMEJ-based donor template with 1 kb homology arms flanked by the H11 safe harbor locus gRNA target site, knock-in rates of 40% of a 5.1 kb bovine sex-determining region Y (*SRY*)-green fluorescent protein (*GFP*) template were achieved in *Bos taurus* zygotes. Embryos that developed to the blastocyst stage were screened for GFP, and nine were transferred to recipient cows resulting in a live phenotypically normal bull calf. Genomic analyses revealed no wildtype sequence at the H11 target site, but rather a 26 bp insertion allele, and a complex 38 kb knock-in allele with seven copies of the *SRY-GFP* template and a single copy of the donor plasmid backbone. An additional minor 18 kb allele was detected that looks to be a derivative of the 38 kb allele resulting from the deletion of an inverted repeat of four copies of the *SRY-GFP* template.

**Conclusion:**

The allelic heterogeneity in this biallelic knock-in calf appears to have resulted from a combination of homology directed repair, homology independent targeted insertion by blunt-end ligation, NHEJ, and rearrangement following editing of the gRNA target site in the donor template.

This study illustrates the potential to produce targeted gene knock-in animals by direct cytoplasmic injection of bovine embryos with gRNA/Cas9, although further optimization is required to ensure a precise single-copy gene integration event.

**Supplementary Information:**

The online version contains supplementary material available at 10.1186/s12864-021-07418-3.

## Background

The targeted integration of large DNA segments into livestock genomes has remained challenging since the production of the first random integrant transgenic livestock were reported 35 years ago [1]. Typically, targeted insertions have been performed in cell lines, followed by somatic cell nuclear transfer cloning (SCNT) [2]. However, SCNT is associated with high rates of both pregnancy and perinatal loss. There are few reports of embryo-mediated targeted insertions in livestock, and they frequently result in mosaic embryos with more than two alleles resulting from independent editing events following the first cleavage division [3]. Mosaic animals are problematic in uniparous large animals with long generation interval (2 years for cattle), as it requires several years to produce a non-mosaic animal through conventional breeding.

Attempts have been made to increase the efficiency of performing targeted gene insertions utilizing the homologous recombination (HR) pathway [4], which is primarily restricted to actively dividing cells (S/G2-phase) and only becomes highly active towards the end of the first round of DNA replication [5]. However, these have been largely unsuccessful in bovine embryos [6], and often result in mosaic animals. A homology mediated end-joining (HMEJ)-based strategy was found to be an efficient gene knock-in strategy in mouse and monkey embryos [7], as well as chicken primordial germ cells [8]. Multiple repair pathways are thought to be involved in mediating a gene knock-in using this method. Previously, we found that the use of a HMEJ repair template to target an insertion to the X chromosome increased the knock-in frequency in bovine embryos as compared to a traditional HR template [9], and that more than a third of knock-in blastocysts analyzed were non-mosaic with precise integrations [10]. Blunt end ligation of cleaved donor template by homology independent insertion was also observed, more frequently in male than female embryos, but no integration of the donor plasmid backbone was ever detected [10].

The objective of this study was to insert a 1.8 kb DNA segment, the *sex-determining region of the Y chromosome* (*SRY)* gene, into a targeted location in the bovine genome. This gene, typically located on the mammalian Y chromosome, is expressed in early embryonic development and results in a cascade of factors necessary for initiating male gonadal development and shutting down development of the female gonad [11]. We wished to investigate whether the inheritance of the bovine SRY gene would be sufficient to trigger the male developmental pathway in XX bovine embryos. Male calves are desirable as sale animals in beef cattle production systems because they have greater feed efficiency than females and reach market readiness at a heavier weight.

Given the time and expense to perform bovine embryo transfers, and the subsequent nine-month gestation required to produce a calf, it was necessary to confirm the presence of the *SRY* insertion prior to embryo transfer to a recipient cow. The diagnostic value of invasive preimplantation biopsies of cells derived from the trophectoderm of blastocysts as a means of screening for knock-ins is decreased in genome edited embryos [12] due to the potential for mosaicism [13]. In the current study a safe harbor locus, H11 on Chromosome 17, was targeted as the insertion site and a fluorescent reporter protein was employed to allow for the non-invasive screening of embryos to identify those carrying the gene insertion prior to embryo transfer.

## Results

### Production of a gene knock-in bull calf

**Fig. 1:**
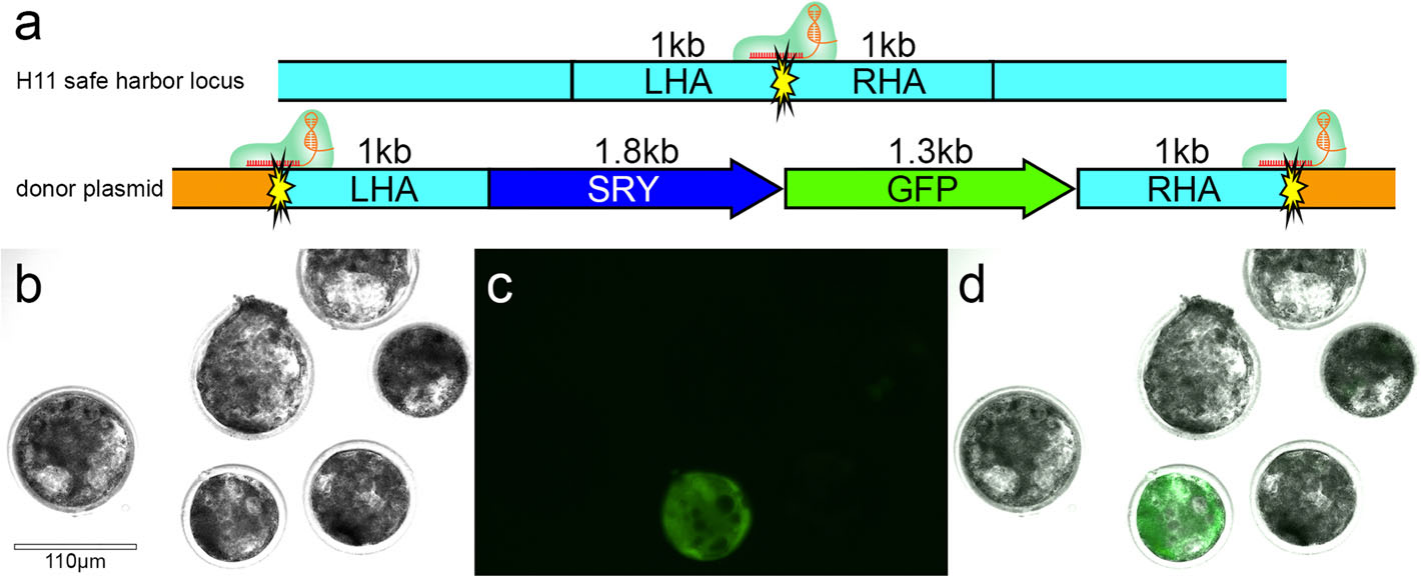
The CRISPR-mediated knock-in of bovine embryos by homology mediated end joining (HMEJ). We utilized the HMEJ donor template design with the green fluorescent protein reporter gene to develop a non-invasive screening method of bovine blastocysts to visualize knock-in embryos. **a** schematic representation of the complete template in the pUC19 plasmid (orange). Yellow starburst = gRNA target site at the H11 locus on chromosome 17 with gRNA/Cas9 ribonucleoprotein complex bound; LHA = left homology arm; SRY = sex-determining region Y; GFP = green fluorescent protein; RHA = right homology arm; kb = kilobase **b** day seven microinjected bovine blastocysts under bright field **c** a filter specific for eGFP fluorescence showing a fluorescent blastocyst, and **d** merge of bright field and fluorescent image

To generate the targeted knock-in bull, a HMEJ donor template containing the 1.8 kb bovine sex-determining region Y (*SRY*) promoter and coding sequencing [14], the 1.3 kb *GFP* reporter transgene coding sequencing with Simian virus 40 (SV40) promoter was designed. It included 1 kb homology arms flanked on the outside by the gRNA target site [15] of the H11 safe harbor locus [16] on bovine chromosome 17 (5.1 kb “complete template”, Fig. 1a). Genomic safe harbors can incorporate exogenous pieces of DNA and permit their predictable function, but these edits do not pose adverse health risks to the host organism [17].

**Fig. 2:**
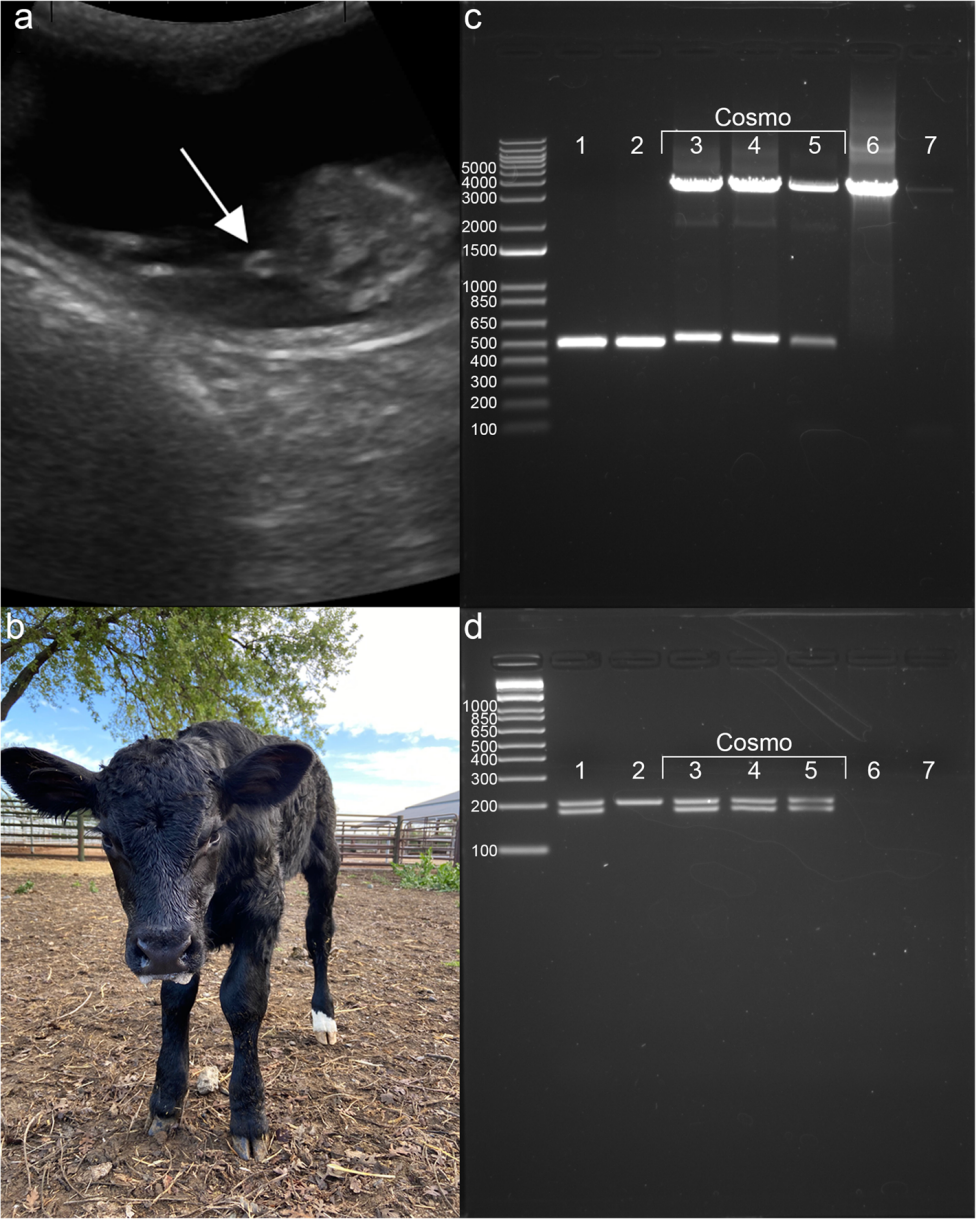
Development of a targeted knock-in bull calf. We monitored and analyzed the development of the *SRY-GFP* knock-in bull calf produced by cytoplasmic injection of a homology mediated end joining donor template and the CRISPR-Cas9 system in bovine zygotes. **a** ultrasound of the day 68 fetus revealing the male genital tubercle (arrow) caudal to the umbilicus indicating a male phenotype, **b** the *SRY-GFP* knock-in bull calf (Cosmo) at 2 days of age, **c** Analysis of *SRY-GFP* knock-in by the polymerase chain reaction (PCR). DNA was extracted from three tissue types: placental cotyledons (trophectodermal origin), blood and fibroblast cells (mesodermal origin). The donor plasmid was used as the positive control and water was used as the negative control. Expected band sizes: wild type 520 bp, *SRY-GFP* knock-in 3721 bp. The lower band from the calf runs higher than wild type due to the 26 bp insertion, and **d** Genotypic sex. Expected band sizes: female 208 bp; male 189 bp & 208 bp. lane 1 = wild type male; lane 2 = recipient female (3113); lane 3 = Cosmo placenta; lane 4 = Cosmo blood; lane 5 = Cosmo fibroblast; lane 6 = plasmid; lane 7 = water

Approximately 200 in vitro fertilized bovine zygotes were microinjected with gRNA/Cas9 ribonucleoprotein complex and HMEJ-template at 6 h post insemination (hpi), which is prior to the initiation of zygote DNA replication at 11–15 hpi. Twenty-two embryos reached the blastocyst stage, and nine (40%) showed green fluorescence indicating successful transgene integration (Figs. 1b-d). These nine embryos were non-surgically transferred to synchronized recipients. The remaining 13 blastocysts were genotyped and sequenced and 11 were found to carry mutations at the H11 locus. One recipient (Tag 3113) was confirmed pregnant by transrectal ultrasonography at day 35 of gestation, and the phenotypic sex was likewise determined at day 68 by the location of the genital tubercle, indicating a male phenotype (Fig. 2a). A healthy 50 kg bull calf was born in April 2020 (Fig. 2b).

DNA was extracted from placenta, calf blood, and the fibroblast cell line derived from the calf, and analyzed for *SRY-GFP* knock-in, as well as genotypic sex. PCR and Sanger sequencing revealed a biallelic edit that included both the complete *SRY-GFP* template and a 26 base pair (bp) insertion into the H11 locus (Fig. 2c), in addition to an XY genotype (Fig. 2d).

### Sequence analysis of the knock-in allele

**Fig. 3:**
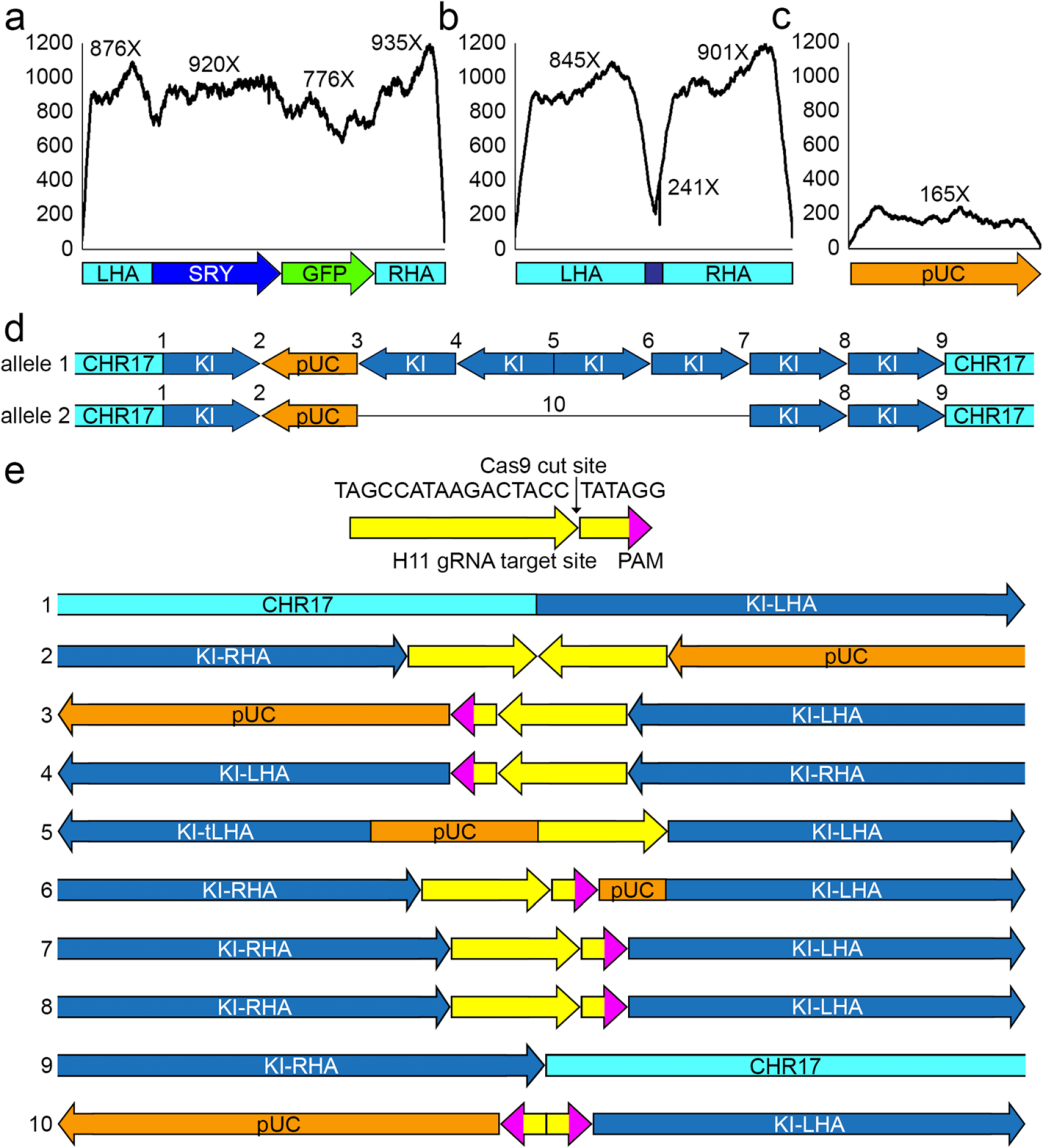
Identification of allelic sequence at the H11 target site. The coverage depth was calculated for the mapped alignment of Illumina NovaSeq whole genome sequencing reads to the expected knock-in, the Sanger sequenced 26 bp knock-in allele, and the pUC19 donor plasmid backbone. Reads were then used to identify the junction sites between the insertions. **a** coverage depth of reads aligned to the complete 5.1 kb *SRY-GFP* template **b** coverage depth of reads aligned to the 26 bp insertion, **c** coverage depth of reads aligned to the 2.7 kb pUC19 donor plasmid backbone (orange), and **d** the 38 kb and 18 kb complex insertions, and **e** gRNA target site and Cas9 cut site (yellow) at the H11 locus on chromosome 17, and schematic representation of the 38 kb and 18 kb complex allele insertion junctions (1–10). LHA = left homology arm; SRY = sex-determining region Y; GFP = green fluorescent protein; RHA = right homology arm; pUC = pUC19 donor plasmid backbone; CHR17 = genomic region outside homology arms on chromosome 17; KI = complete 5.1 kb *SRY-GFP* template; tLHA = truncated left homology arm; PAM = protospacer adjacent motif

Given that the PCR results from the samples taken from tissue types of trophectodermal and mesodermal origin were identical, DNA extracted from blood was used for Illumina whole-genome sequencing (paired-end, 150 bp) on a NovaSeq 6000 sequencer (Novogene, USA) to 268X coverage. Raw reads were mapped to the complete template on chromosome 17 (Fig. 3a), the 26 bp insertion allele (Fig. 3b), and the HMEJ donor pUC19 plasmid backbone (Fig. 3c). There was a 4X increase in reads that aligned to the complete template compared to the 26 bp insertion. In addition, some reads aligned to the pUC19 plasmid backbone (Fig. 3c). This suggested integration of the donor plasmid backbone, in addition to the intended knock-in template, as was observed previously [18, 19].

To investigate the insertions more fully, PacBio long-read sequencing was generated from the same blood sample. From all PacBio reads, we identified 314 sequences with some similarity to the complete template, the 26 bp insert, the donor plasmid backbone, and/or the H11 locus on chromosome 17. Then, a reference sequence was generated which included the complete ARS-UCD1.2 bovine genome sequence [20], the plasmid backbone and the complete template sequences. Mapping the 314 candidate reads, we detected no wild-type H11 allele and 3 insertion alleles. The 26 bp insertion into the wild-type H11 allele that was detected by Sanger sequencing (Fig. 3b) was supported by 49 long reads. The other 2 alleles each included 1 copy of the plasmid backbone sequence (Fig. 3c) with multiple copies of the complete template (Fig. 3a, d). The larger ~ 38 kb allele had around 50X coverage and consisted of 7 copies of the complete template along with 1 copy of the plasmid backbone. The smaller 18 kb allele had 3 copies of the complete template in addition to 1 copy of the plasmid backbone and was unambiguously supported by only 5 long reads. This allele is identical to the larger complex allele but missing the middle 4 copies of the complete template sequence (Fig. 3d).

### Fluorescence in situ hybridization (FISH) of *SRY*

**Fig. 4:**
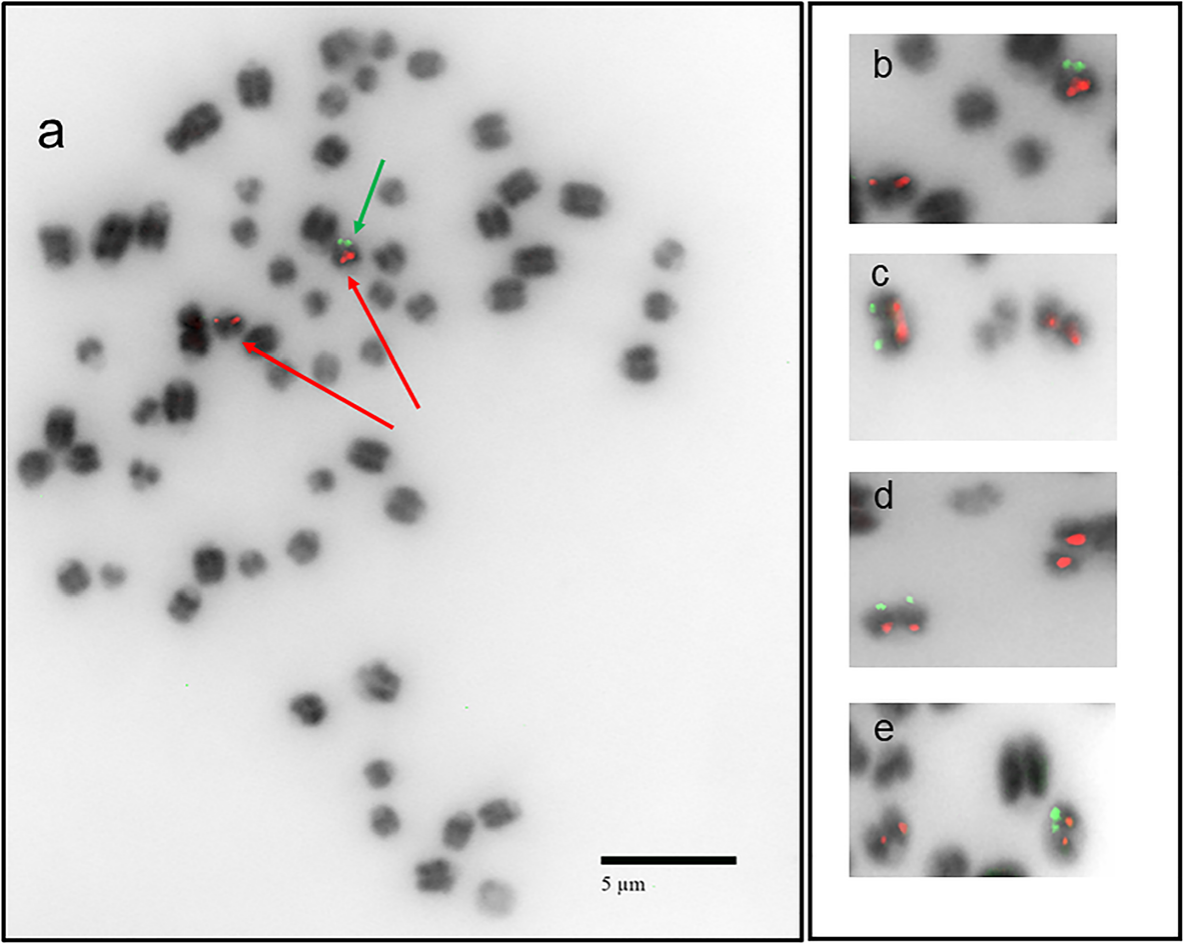
One BTA 17 homolog identified as the map location for the SRY insert in the CRISPR-targeted knock-in bull calf by dual color fluorescence in situ hybridization (FISH). FISH with the donor plasmid (SRY-GFP Anti-Digoxigenin-Fluorescein) as the probe identified one acrocentric chromosome with a positive signal at the q-arm terminal region confirming a single insertion site into the knock-in calf genome. The acrocentric was identified as BTA 17 utilizing a chromosome-specific centromere-proximal probe labelled with Red dUTPs (CHORI BAC 371i17, see Methods). The q-arm terminal location of the SRY green signal found opposite to the centromere proximal BAC red signal compliments the expected insertion location at the safe-harbor as per the sequencing results. Male and female controls (no insertion) were also examined using the same SRY probe with no signal(s) observed (data not shown). **a** Diploid mitotic metaphase chromosome spread from a fibroblast culture derived from the SRY-GFP knock-in bull shows a normal karyotype, 2n = 60 with a single SRY-GFP positive signal (green arrow) on one of the two BTA17 chromosomes (red arrows) **b** enlarged SRY-GFP knock-in BTA 17 chromosome (red and green signals) along with the other BTA 17 chromosome (red signal only) from cell depicted in (**a**) and **c, d, e** enlarged BTA 17 chromosomes from other cells illustrate the reproducibility of the FISH results. Chromosomes shown in b-e were all enlarged to the same degree

The *SRY* insert was consistently detected near the q arm terminus of one chromosome providing additional evidence for the insertion into a single location (Fig. 4). The chromosome size and type, i.e., smaller-sized acrocentric, align with that expected for *Bos taurus* (BTA) chromosome 17 and this insert map location was cytogenetically confirmed by dual color FISH experiments employing a BTA 17 specific BAC. The *SRY* signal detected at the knock-in location was likely amplified given the presence of multiple copies of the gene inserted at the H11 target site as shown by sequencing. Conversely, a faint *SRY* signal was only occasionally detected on the Y chromosome, and only following significant signal amplification by image analysis. This result is likely due to the non-repetitive and small size of the single copy SRY gene in its native state, coupled with the resolution-scale of FISH.

## Discussion

The birth of this calf represents the first successful targeted integration of a large DNA segment produced by embryo-mediated genome editing in cattle. Although we achieved a 40% knock-in rate as determined by GFP expression in blastocysts, only 22 (11%) of the ~ 200 microinjected embryos developed to the blastocyst stage. We previously observed a significant reduction in blastocyst development following microinjection of editing regents into MII oocytes (10.2%) and presumptive zygotes 6 hpi (17.6%), as compared to non-injected controls (29.3%) [10, 15]. Additionally, only one of the nine embryos transferred resulted in a live birth. This is a low success rate (11.1%), although it is a small sample size, and on average only 27% of recipients receiving conventional in vitro produced (IVP) embryos result in a live calf [21]. Further experiments with a larger number of embryos will be also be required to determine if briefly screening blastocysts for GFP affected viability. It is known that UV light can be harmful to living embryos, although others have reported viable pregnancies following short exposure of bovine blastocysts to blue light to screen for GFP expression [22]. More generally, the current low efficiencies of precise targeted integration of large DNA segments, embryo development and live births of non-mosaic animals limits the utility of embryo-mediated gene knock-ins in cattle breeding programs [6, 23].

The only other group to report a bovine embryo-mediated targeted gene knock-in used TALENs and a single-strand oligonucleotide (ssODN) HR donor template to introduce a targeted 9 bp deletion in the bovine lactoglobulin (*LGB*) gene [24]. In that experiment, the editing reagents were introduced into 1511 bovine zygotes at 18 hpi. Of these, 234 (15%) developed to grade 7 or 8 embryos, of which 50 (21%) were confirmed to carry the 9 bp *LGB* deletion by biopsies of 10–15 cells derived from the trophectoderm of blastocysts. Of these, 13 were transferred to generate three (23%) live births, of which one calf died shortly after birth.

In the current experiment, no H11 wild-type allele was amplified by PCR (Fig. 2c). There were 11 short read sequences (6 after deduplication) that supported H11 wild-type sequence in the more than 250X sequence coverage, but no single long read contained wild-type H11 sequence. The 26 bp and 38 kb insertion alleles were both represented at around 50X coverage in the long-read sequencing data. Collectively, these data suggest that a biallelic edit at the zygote stage, one of which was repaired by NHEJ resulting in a 26 bp insertion, and the other 38 kb complex allele knock-in which appears to have resulted from a combination of homology directed repair, homology independent targeted insertion by blunt-end ligation, and rearrangement following editing of the gRNA target site in the donor template.

Multiple copies of the complete template in both forward and reverse orientations in the 38 kb allele was not expected with the HMEJ-mediated strategy, and was not observed in our previous study using this approach [10]. Such concatenation is more typical of homology independent targeted insertion (HITI) [25]. In the case of this bull calf, it appears the far left and right homology arms were repaired by HR as there is no H11 gRNA target site footprint at the boundary where the left homology arm meets the 5′ wild-type genomic sequence of bovine chromosome 17, or where the right homology arms meets the 3′ wild-type sequence. Many of the other junctions between inserts contain partial H11 gRNA target sequences (Fig. 3e). This suggests that the RNP complex cut the donor plasmid at the H11 gRNA target sites and the resulting double-stranded fragments integrated by blunt end ligation. The repair mechanism for junctions 5 and 6 in the 38 kb complex allele is less apparent as both sequences include a short plasmid backbone sequence, 56 bp and 9 bp, respectively. The overall complexity of this insertion allele suggests a potential concern associated with knock-in strategies which involve flanking the homology arms with sgRNA target sites. In this study it appears Cas9 cleavage of these target sites contributed to the integration of the multiple copies of the donor template in various orientations, and one copy of the plasmid backbone, rather than the precise integration that was predicted.

A minor 18 kb complex allele was also detected at approximately one tenth the read coverage of the 38 kb allele. The only difference in the DNA sequence between the 38 kb and 18 kb complex alleles was the loss of four complete templates (two in the reverse direction and 2 in the forward direction) (Fig. 3d). This indicates that the 18 kb allele, which was present in all 3 tissue types analyzed (placenta, fibroblasts, and blood; data not shown), may represent a deletion derivative of the larger 38 kb allele, rather than a separate editing event. The inverted repeat nature of the sequence that was deleted, i.e., two complete *SRY-GFP* templates in the reverse direction followed immediately by two complete *SRY-GFP* templates in the forward direction, may indicate instability in the 38 kb insertion allele.

While multiple copies of the donor template and a single copy of the donor backbone were inserted into the target location, there was no off-target insertion of the donor template or donor backbone detected. This was demonstrated by the lack of short or long reads containing donor template or donor backbone sequence in any region outside the H11 locus. In addition, the only FISH signals detected for the presence of the insert were that of a single homolog at the q-arm end of BTA 17, which aligns with the H11 locus which is located ~ 3 kb from the terminus of BTA 17.

Strategies aimed at avoiding unwanted plasmid backbone integration in genome editing include using single stranded DNA (ssDNA) repair templates, which have a significantly reduced frequency of unintended genomic integration. However, the primary success with targeting a knock-in of embryos using ssDNA has been through attempting allelic conversions, such as small insertions, deletions or single nucleotide polymorphisms. Each of these cases was performed using ssODNs of varying length ranging from 35 to 120 bp [26–28]. The largest integration performed using ssDNA was a 1368 bp insert using a ~ 1.5 kb ssODN in mouse embryos in a method called Easi-CRISPR [29]. Attempts to insert larger segments of DNA using ssODN through microinjection or electroporation have been unsuccessful in embryos [30].

In this experiment we chose to use a fluorescent marker to identify blastocysts with the *SRY* knock-in, and to target an autosomal safe harbor locus following our previous unsuccessful attempts to obtain live calves when targeting the *SRY* to a X chromosome locus and screening for knock-ins using embryo biopsy [10]. It is possible that the inclusion of the SV40 promoter to drive the expression of the *GFP* gene could result in the silencing of the adjacent *SRY* gene, as has been commonly observed with the hypermethylation of the cytomegalovirus (CMV) promoter. The SV40 promoter has been found to maintain more steady levels of expression when stably integrated in mammalian cells as compared to the CMV promoter [31]. A recent paper reported transgenic cattle expressing GFP driven by the human elongation factor 1α promoter showed stable GFP expression over 6 years and F2 germline transmission without gene silencing [32]. It is also possible that the presence of multiple copies of the transgene in the complex alleles in the current study may also lead to repeat-induced gene silencing.

Although the addition of the *GFP* gene technically made the knock-in bull calf transgenic, the United States Food and Drug Administration regulates all genomic alterations in animals as new animal drugs [33], irrespective of whether a transgene is present [34]. As we had no intention for this genetically altered research line to enter the food chain, the inclusion of the *GFP* transgene in the donor template design to provide a rapid, non-invasive screening method to ensure that only knock-in embryos were transferred to recipient cows, outweighed the fact that it was a transgene.

## Conclusions

The low efficiency of direct HR repair in zygotes, especially for the introduction of large DNA sequences, remains an obstacle for the incorporation of useful genetic variants into livestock genetic improvement programs. The HMEJ-based strategy used in this study did increase the efficiency of HR editing in zygotes, but it also resulted in multiple homology independent blunt-end insertions, including one copy of the donor plasmid backbone. Unintended homology independent insertions may not be problematic for some research applications; however this potential is untenable for embryo-mediated therapeutic applications where precise integration is requisite, and would also pose potential challenges for the regulatory approval of food animal applications.

## Methods

### Experimental design

The objective of this study was to produce a targeted gene knock-in *Bos taurus* bull by direct cytoplasmic microinjection of single-cell bovine embryos using a donor template containing the bovine *SRY* promoter and coding sequence, the *gfp* coding sequence with SV40 promoter utilizing the HMEJ-approach. Once a pregnancy was established, the phenotypic sex was determined by transrectal ultrasound and following birth, genotypic sex was determined, and the on-target and off-target integration of the donor template was evaluated using short and long read whole genome sequencing technology.

### Embryo production

Ovaries were obtained from cull *Bos taurus* cows of unknown breed at a local processing plant and transported in warm sterile saline at temperature of 35–37 °C. Oocyte-cumulus-complexes (COCs) were aspirated from follicles using a vacuum aspiration system and cultured in groups of 50 COCs in 500 μL of BO-IVM culture media (IVF Biosciences, Falmouth, UK) for 18 h at 38.5 °C in a humidified 5% CO_2_ incubator. COCs were then washed and transferred in groups of 25 to 60 μL drops of SOF-IVF media [35] with 2 × 10^6^ sperm per mL and covered in mineral oil. Sperm and COCs were incubated for 6 h at 38.5 °C in a humidified 5% CO_2_ incubator. Presumptive zygotes were then denuded by light vortex and transferred to 25 μL of BO-IVC culture media (IVF Biosciences, Falmouth, UK). Embryos were cultured for 7 days at 38.5 °C in a humidified atmosphere of 5% CO_2_, 5% O_2_, and 90% N_2_.

### Guide-RNA and donor plasmid construction

The guide-RNA (gRNA) targeting the H11 safe harbor locus on bovine chromosome 17 was designed as previously described [15] (TAGCCATAAGACTACCTAT) and commercially synthesized (Synthego, Redwood City, CA, USA). The donor plasmid construct was designed as previously described [9], containing the endogenous bovine sex-determining region Y, (*SRY*) promoter and coding sequence [14], the green fluorescent protein (*GFP*) coding sequence and SV40 promoter, and 1 kb homology arms flanked on either side by the CRISPR target site (Fig. 1a). Each piece was commercially synthesized (GeneWiz, LLC, South Plainfield, NJ, USA) and inserted into a pUC19 plasmid using Gibson Assembly Master Mix (New England Biolabs, Inc., Ipswich, MA). Plasmids were clonally amplified using 5-alpha Chemically Competent *E. coli* (High Efficiency) (New England Biolabs, Inc., Ipswich, MA) and extracted using the EndoFree Plasmid Maxi Kit (Qiagen, Inc., Valencia, CA).

### Embryo injection and evaluation

Approximately 200 in vitro fertilized bovine zygotes were injected approximately 6 h post insemination using laser assisted cytoplasmic injection [36] with 6 pL of solution containing 67 ng/μL of synthetic gRNA, 167 ng/μL of Cas9 protein (PNA Bio, Inc., Newbury Park, CA) and 133 ng/μL of donor plasmid. Embryos were then cultured in BO-IVC culture media (IVF Biosciences, Falmouth, UK) for 7 days at 38.5 °C in a humidified atmosphere of 5% CO_2_, 5% O_2_, and 90% N_2_. On day seven, embryos were scored for developmental stage reach and 22 high grade seven blastocysts were selected and analyzed using fluorescent microscopy on a Nikon Eclipse TE2000-U advanced inverted epifluorescence microscope at 20X magnification using a filter specific for eGFP fluorescence. Fluorescent images of *GFP* expressing blastocysts were taken using an Echo Revolve 4 upright, inverted, brightfield microscope at 10X magnification using transillumination for bright field and FITC for *GFP* expression.

### Embryo transfer

Estrus synchronization was initiated in 15 nulliparous heifers from the Department of Animal Science, University of California, Davis commercial cow herd by inserting an intravaginal progesterone device (1.38 g; Eazi-Breed CIDR; Zoetis) and intramuscular administration of gonadorelin (100 mcg; Factrel; Zoetis) on day 0 (16 days prior to transfer). This number of recipients was chosen with the objective of obtaining at least two SRY knock-in bull calves assuming 60% response to synchronization, and an expectation that 27% of recipients receiving conventional in vitro produced (IVP) would result in a live calf. On day 7, the CIDR was removed and intramuscular prostaglandin (25 mg; Lutalyse; Zoetis) was administered. Recipients were monitored for estrus, and a second intramuscular dose of gonadorelin (100 mcg; Factrel; Zoetis) was administered on day 9. Prior to transfer on day 16, recipient response to synchronization was confirmed via detection of an appropriate corpus luteum with transrectal ultrasonography and nine recipients were deemed suitable for embryo transfer. Prior to transfer, each recipient received a caudal epidural using 100 mg 2% lidocaine (Xylocaine; Fresenius). A total of nine embryos were transferred via non-surgical, transcervical technique, with each GFP positive blastocyst being deposited into the uterine horn ipsilateral to the corpus luteum. Pregnancy was diagnosed on day 35 of embryonic development by transrectal ultrasonography (5.0 MHz linear probe; EVO Ibex, E.I. Medical Imaging), and sex was likewise determined at day 68 of development. A single knock-in bull calf was born in April, 2020, and was monitored and maintained at the University of California, Davis Beef Cattle Barn under normal husbandry conditions. The bull calf remains at this facility while he develops to sexual maturity.

### DNA extraction and PCR analysis

Whole blood (5 ml) was collected in EDTA vacutainers (Becton Dickinson) by a veterinarian from the UC Davis veterinary hospital large animal clinic. DNA was extracted either from the buffy coat using the DNeasy Blood and Tissue kit (Qiagen, Inc., Valencia, CA, USA) or from whole blood using red blood cell lysis, SDS/Proteinase K cell lysis, phenol/chloroform/isoamyl alcohol clean up and ethanol precipitation. The placental cotyledon was collected and cut into small pieces. DNA was then extracted from 25 mg of placental cotyledon using the DNeasy Blood and Tissue kit. An ear punch biopsy was taken from the bull calf and used to establish a fibroblast line in culture. Cells were passaged twice in DMEM media (Thermo Fisher, Waltham, MA, USA) containing 10% fetal bovine serum (Thermo Fisher, Waltham, MA, USA), 1% Glutamax (Thermo Fisher, Waltham, MA, USA) and 1% penicillin/streptomycin (Thermo Fisher, Waltham, MA, USA). After the second passage, cells were collected and DNA was extracted using the DNeasy Blood and Tissue kit (Qiagen Inc., Valencia, CA, USA). The target regions were amplified using the polymerase chain reaction (PCR) using primers (F – CCCCAGTGTTGTGCATGTAG; R – GTGAATGCCACTGCTGTGTT) for the H11 locus [15] and primers (F – AGGAAGCCAGGAAAGTAA; R – CATCCACGTTCTAAGTCTC) for genotypic sexing. The knock-in PCR was performed on a SimpliAmp Thermal Cycler (Applied Biosystems, Foster City, California, USA) with 12.5 μL LongAmp *Taq* 2X Master Mix (New England Biolabs, Inc., Ipswich, MA), 9.5 μL of H_2_O, 1 μL of each primer at 10 mM and 1 μL of DNA for 5 min at 94 °C, 35 cycles of 30 s at 94 °C, 30 s at 60 °C and 4 min at 65 °C, followed by 15 min at 65 °C. The sexing PCR was performed on a SimpliAmp Thermal Cycler (Applied Biosystems, Foster City, California, USA) with 12.5 μL GoTaq Green Master Mix (Promega Biosciences, LLC, San Luis Obispo, CA, USA), 9.5 μL of H_2_O, 1 μL of each primer at 10 mM and 1 μL of DNA for 5 min at 94 °C, 35 cycles of 30 s at 94 °C, 30 s at 55 °C and 30s at 72 °C, followed by 5 min at 72 °C. Products were visualized on a 1% agarose gel using a ChemiDoc-ItTS2 Imager (UVP, LLC, Upland, CA), purified using the QIAquick Gel Extraction Kit (Qiagen, Inc., Valencia, CA) and Sanger sequenced (GeneWiz, South Plainfield, NJ).

### Whole genome sequencing and mapping

Genomic DNA extracted from the buffy coat was submitted to Novogene for library construction and whole genome sequencing. Samples were sequenced on an Illumina NovaSeq 6000 sequencer with paired end, 150 bp reads. Raw reads were aligned to the donor plasmid backbone, as well as the predicted knock-in map using Bowtie2-default v2.3.4.1. SAM files were converted to BAM files, sorted and indexed using SAMtools v1.12.0 [37]. Depth was called at each base along the alignment using SAMtools depth v1.12.0 [37].

### Assessment of long reads

Genomic DNA extracted from whole blood was submitted for PacBio long read sequencing in a Sequel II SMRTcell (GeneWiz, USA). Input bam files were converted into FASTA files then assembly-stats (https://github.com/sanger-pathogens/assembly-stats.git) was used to assess the size of the reads. There were 19,709,419 reads with total sum length 292,074,095,630 bp which is ~97x coverage of the bovine genome. The average read length was 14,819.01 bp while the largest read length was 249,262 bp.

### Identification of candidate long reads

To identify the reads that had any similarity to the possible inserts, cloning plasmid and/or insertion locus on chromosome 17, a bait file was generated containing:

- The wild-type locus on Chr17 (1 kb before and after the break point).
- The pUC19 plasmid backbone.
- The originally proposed knock in sequence including the homology arms (complete template).
- The 26 bp allele detected by Sanger sequencing.

Alignment of all input long reads against the bait file using BLASR (5.3.3-SL-release-8.0.0) recruited 314 reads.

### Identification of possible structures of edited alleles

To find out the structure of any allele connecting the introduced sequences to the bovine chromosomes, a new reference was generated to include the bovine reference ARS-UCD1.2 [20], together with the pUC19 plasmid and complete template sequences. All candidate reads were aligned against the new reference. To enable better delineation of the allele structure, each read was fragmented into 1 kb subreads with 0.5 kb overlap. Read alignments were tested manually and classified into groups that support each allele structure. All suggested structures supported with at least two reads from more than one cluster were considered for further analyses.

### Identification of possible junctional sequences between the allele blocks

The last 50 nucleotides of each edge from the plasmid and proposed complete template were converted into overlapping k-mers of 25 bp length. This way each 50 bp edge was transformed into 26 k-mers. Any short read containing at least one of these k-mers or their reverse complement were selected. The k-mer selected reads (660 reads covering the plasmid edges and 3246 reads covering the complete template edges) were error trimmed using Khmer 2 software package. Trimmed reads were assembled into contigs using SSAKE. Contigs were annotated by BLAST alignment against plasmid and complete template sequences and manual examination to identify the novel junction sequences.

### Confirmation of the allele sequences

To further confirm the exact sequence of the three putative alleles, long reads covering each sub-structures of the proposed alleles were subjected to multiple sequence alignment using MAFFT (http://europepmc.org/article/MED/30976793). To improve the quality of alignment, most of the wild-type sequences were trimmed from the reads. The aligned sequences were used to generate a consensus sequence using the cons tool of EMBOSS package. The consensus sequence was re-aligned using BLAST to the nr database or the proposed sequences. The full sequence of the three insertion alleles and surrounding bovine genomic sequence are included in Supplementary Materials.

### Fluorescence in situ hybridization

The fibroblast line derived from an ear punch biopsy taken from the bull calf was plated in a T75 flask in DMEM media (Thermo Fisher, Waltham, MA, USA) containing 10% fetal bovine serum (Thermo Fisher, Waltham, MA, USA), 1% Glutamax (Thermo Fisher, Waltham, MA, USA) and 1% penicillin/streptomycin (Thermo Fisher, Waltham, MA, USA). Once the cells reached ~ 80% confluency, 1.25% Gibco KaryoMAX Colcemid Solution (Thermo Fisher, Waltham, MA, USA) was added to the media and incubated at 37 °C and 5% CO_2_ for 1 h. Cell were then collected, resuspended in 10 mL of 0.56% KCl hypotonic solution and incubated at 37 °C for 10 min. Cells were then fixed in a 3:1 methanol to glacial acetic acid solution at 4 °C for 1 week. Once fixed, three to four drops of cell solution were applied to slides at a 45° angle and allowed to air dry. Slides were then hardened, i.e., aged at − 20 °C for 1 week. Slides were then used for fluorescence in situ hybridization as previously described [38, 39] using Roche DIG-Nick Translation Kit and Anti-Digoxigenin-Fluorescein, Fab fragments (Roche Applied Science, Upper Bavaria, Germany) to label the plasmid containing SRY. The BTA 17 chromosome-specific BAC (CHORI 371i17) with a centromere proximal location (15,482,193 – 15,677,551) was labelled using Red dUTP (Abbott Molecular, Des Plaines, IL) and a direct Nick Translation Kit (Abbott Molecular). Chromosomes were visualized using Vectashield mounting media with DAPI (Abcam, Cambridge, MA, USA). Mitotic metaphase chromosomes were examined, and images collected using an Olympus BX41 epifluorescence microscope equipped with an automatic filter wheel (Chroma Technology 82,000, DAPI/FITC/TRITC filter set), X-cite 120 Series metal-halide fiber optic lamp and Applied Imaging software (CytoVision version 7.4 GENUS, Leica Biosystems).

## Supplementary Information

**Additional file 1.**

**Additional file 2.**

## Data Availability

Raw sequence reads from Illumina NovaSeq and PacBio Sequel II sequencing are available in the NCBI Sequence Read Archive as BioProject PRJNA625124, BioSample SAMN14593732 and SRA accession number SRR12005315 – SRR12005330.
