# Supplementary figures and images for: One-step generation of a targeted knock-in calf using the CRISPR-Cas9 system in bovine zygotes
Source: BMC Genomics. 2021 Feb 12;22:118. doi: 10.1186/s12864-021-07418-3 (PMC7881600; doi:10.1186/s12864-021-07418-3)

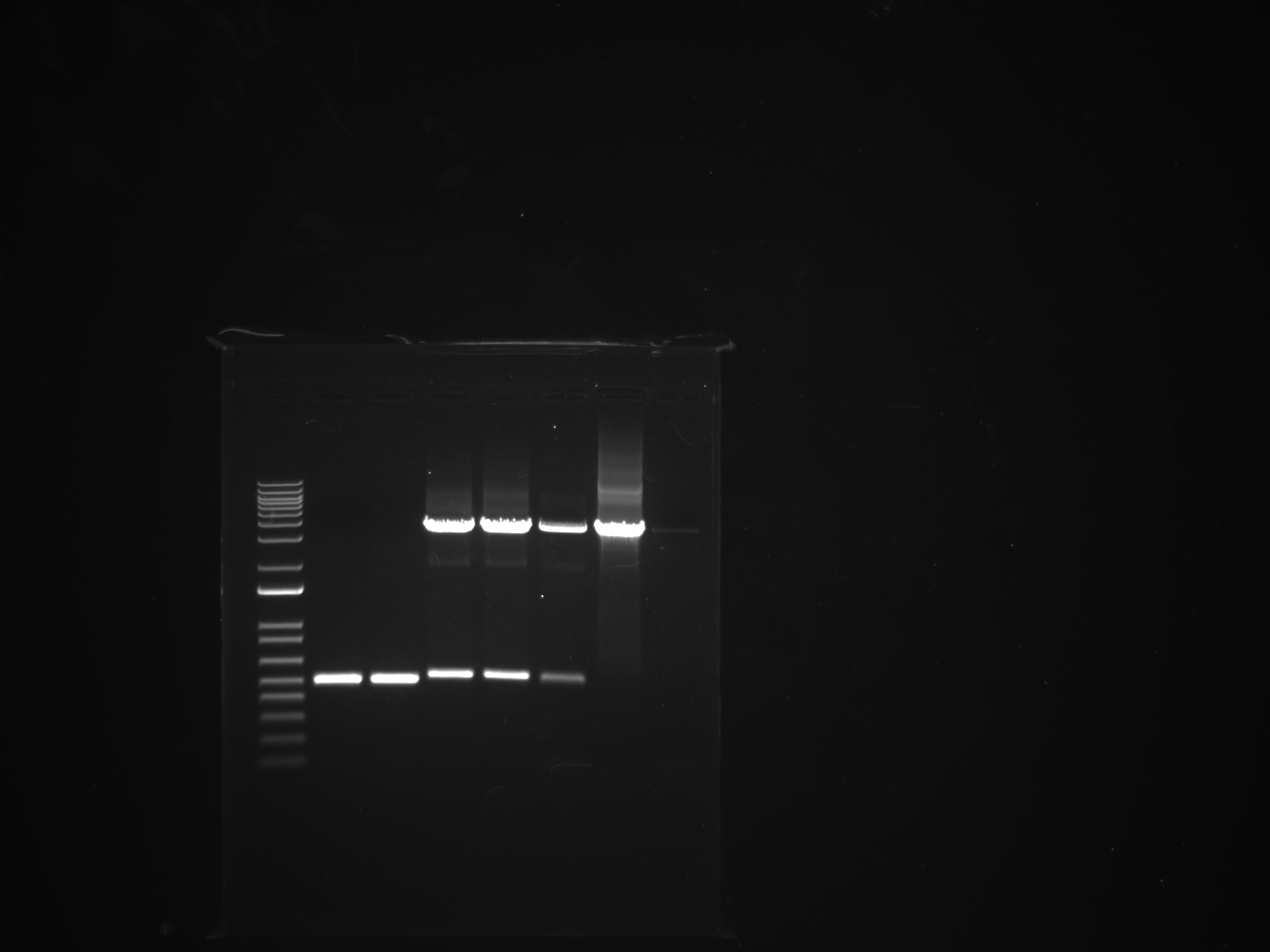

Supplement: Supplementary file 1 — Additional file 1. [file 12864_2021_7418_MOESM1_ESM.tif]

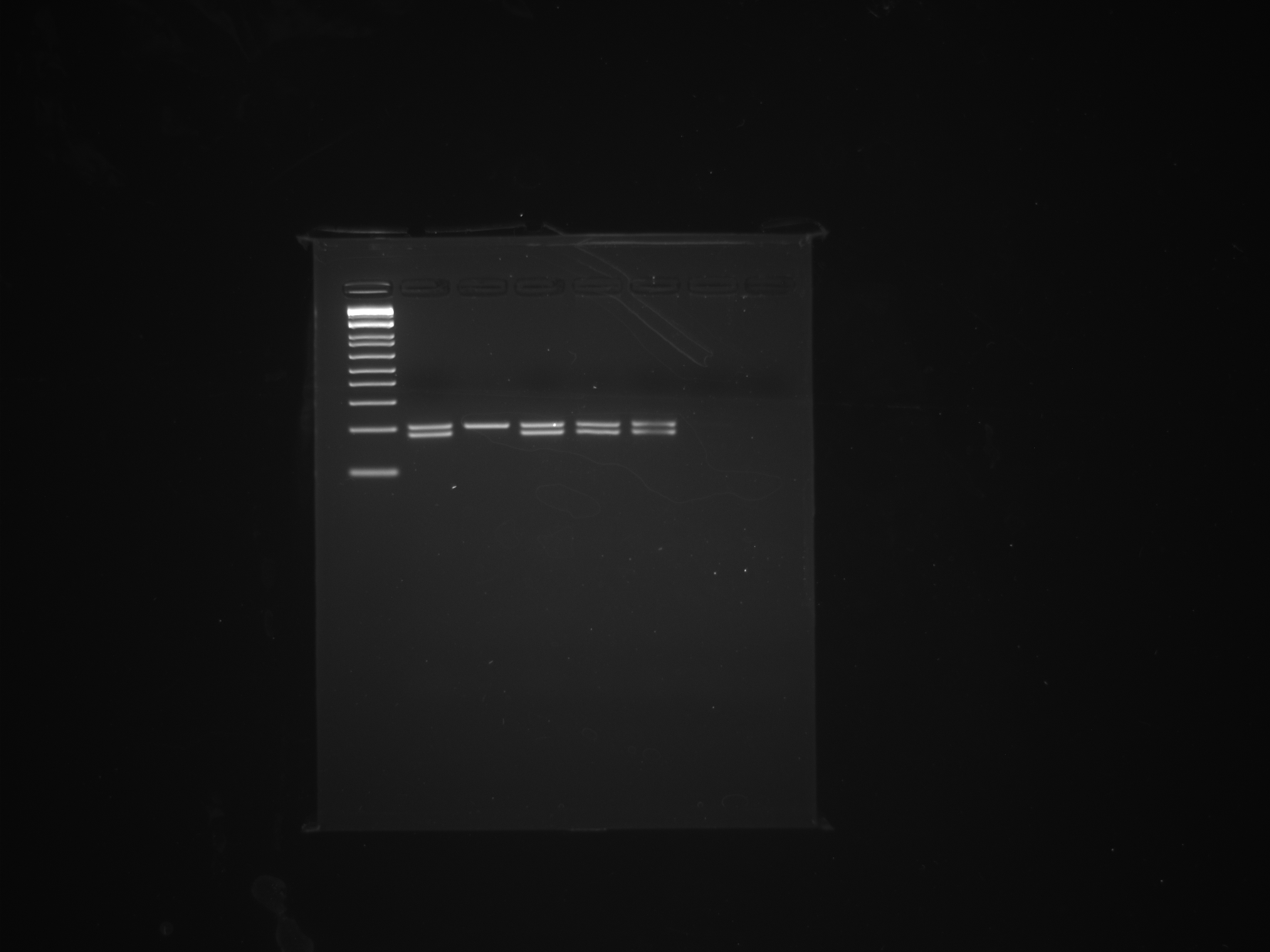

Supplement: Supplementary file 2 — Additional file 2. [file 12864_2021_7418_MOESM2_ESM.tif]
